# Vascular and hormonal interactions in the adrenal gland

**DOI:** 10.3389/fendo.2022.995228

**Published:** 2022-11-24

**Authors:** Alaa B. Abdellatif, Fabio L. Fernandes-Rosa, Sheerazed Boulkroun, Maria-Christina Zennaro

**Affiliations:** ^1^ Université Paris Cité, PARCC, INSERM, Paris, France; ^2^ Assistance Publique-Hôpitaux de Paris, Hôpital Européen Georges Pompidou, Service de Génétique, Paris, France

**Keywords:** adrenal gland, aldosterone, vascularization, primary aldosteronism (PA), aldosterone producing adenoma

## Abstract

Primary aldosteronism is the most common form of secondary arterial hypertension, due to excessive aldosterone production from the adrenal gland. Although somatic mutations have been identified in aldosterone producing adenoma, the exact mechanisms leading to increased cell proliferation and nodule formation remain to be established. One hypothesis is that changes in vascular supply to the adrenal cortex, due to phenomena of atherosclerosis or high blood pressure, may influence the morphology of the adrenal cortex, resulting in a compensatory growth and nodule formation in response to local hypoxia. In this review, we will summarize our knowledge on the mechanisms regulating adrenal cortex development and function, describe adrenal vascularization in normal and pathological conditions and address the mechanisms allowing the cross-talk between the hormonal and vascular components to allow the extreme tissue plasticity of the adrenal cortex in response to endogenous and exogenous stimuli. We will then address recent evidence suggesting a role for alterations in the vascular compartment that could eventually be involved in nodule formation and the development of primary aldosteronism.

## Introduction

The adrenal gland is an endocrine tissue composed of two distinct zones with different functions: the cortex, responsible for steroid biosynthesis, and the medulla, where catecholamine biosynthesis occurs. In human, the adrenal cortex is subdivided into three distinct functional zones: the outer part of the adrenal cortex is formed by the zona glomerulosa (ZG) responsible for mineralocorticoid biosynthesis, the intermediate and thickest part is formed by the zona fasciculata (ZF), responsible for glucocorticoids biosynthesis, and the inner part is formed by the zona reticularis (ZR) responsible for the biosynthesis of adrenal androgens (1). Both adrenal glands receive arterial blood supply from the ventral aorta and the renal artery; the left adrenal is also supplied by the caudal branch of the aorta and the right adrenal by the phrenic artery (2)

Interestingly, the adrenal gland is one of the most vascularized organs. It has been shown that this organized vascular network played an important role during embryogenesis to ensure adrenal growth and differentiation, but also during whole life to provide precursors necessary for the biosynthesis of steroid hormones and to allow their secretion in blood flow (3). The specific ramification of adrenal cortex vasculature suggests strong interactions between endothelial and adrenal cells (4), allowing their coordinated development. Moreover, the proximity between endothelial and endocrine cells allows a rapid release of steroids into the blood flow.

Primary aldosteronism (PA) is the most common form of secondary arterial hypertension due to autonomous aldosterone production from the adrenal cortex. The two major causes are unilateral aldosterone producing adenoma (APA) or bilateral adrenal hyperplasia (BAH, also called idiopathic hyperaldosteronism). Patients show increased blood pressure, often associated with hypokalemia. Diagnosis is made in the presence of suppressed renin levels and increased aldosterone to renin ratio and is confirmed by one of different suppression tests. Adrenal imaging and adrenal vein sampling allow to distinguish between unilateral and bilateral forms and to introduce optimal treatment, either adrenalectomy for APA or treatment with mineralocorticoid receptor antagonists for bilateral forms (5). PA is found in up to 10% of patients with hypertension (6, 7) and its prevalence increases with the severity of hypertension (8). Over the past ten years, major progress has been made in elucidating genetic defects underlying familial and sporadic forms of PA. In particular, somatic mutations have been identified in genes coding for ion channels (*KCNJ5*, *CACNA1D*, *CACNA1H*, *CLCN2*) and ATPases (*ATP1A1*, *ATP2B3*) in up to 96% of APA (9–13). These mutations lead to cell membrane depolarization (*KCNJ5*, *ATP1A1*, *CLCN2*) or increase intracellular calcium (*ATP2B3*, *CACNA1D*, *CACNA1H*), leading to activation of calcium signaling that is the main trigger for aldosterone biosynthesis. In addition, mutations in *CTNNB1* coding for β-catenin, à key regulator of adrenal cortex development and function, have been identified in a subset of patients with PA, either alone or in association with mutations of *GNAQ*/*GNA11* in patients presenting with PA at puberty, pregnancy or menopause (14). However, it is still unclear, whether those mutations, in addition to promoting aldosterone biosynthesis, also increase cell proliferation and nodule formation and different hypotheses have emerged in recent years. Among them it has been postulated that changes in vascular supply to the adrenal cortex, due to phenomena of atherosclerosis or high blood pressure, may influence the morphology of the adrenal cortex, resulting in a compensatory growth and nodule formation in response to local hypoxia (15). This could eventually lead to the development of APA in extreme cases.

In this review we will briefly summarize the mechanisms regulating aldosterone biosynthesis in the adrenal gland, describe adrenal vascularization in normal conditions and how the cross-talk between the hormonal (epithelial) and vascular (endothelial) components ensures adrenal cortex growth and function under physiological conditions. We will then address recent evidence suggesting a role for alterations in the vascular compartment that could eventually be involved in nodule formation and the development of PA.

## Regulation of aldosterone biosynthesis in the adrenal cortex

Aldosterone is synthesized in the ZG of the adrenal cortex from the precursor cholesterol through a series of enzymatic steps involving in particular the enzyme aldosterone synthase, encoded by *CYP11B2*, which is specifically expressed in this zone. Regulation of aldosterone biosynthesis is aimed at maintaining its essential functions as one of the principal regulators of extracellular fluid and electrolyte homeostasis as well as blood pressure, due to its effects on sodium reabsorption and potassium secretion in the kidney. Thus, aldosterone biosynthesis is regulated by the renin-angiotensin system (RAS), potassium concentrations and, to a lesser extent, by the adrenocorticotropic hormone (ACTH) (1). Following dehydration or salt loss, activation of the RAS regulates aldosterone biosynthesis *via* angiotensin II (AngII) binding to its type 1 receptor (AT1R) in ZG cells. This activates the inositol triphosphate pathway that stimulates Ca^2+^ release from the endoplasmic reticulum; alternatively, AngII inhibits potassium channels and the Na^+^,K^+^-ATPase, inducing cell membrane depolarization, followed by opening of voltage-gated calcium channels. Both pathways increase intracellular calcium concentrations and activate calcium signaling, which regulates different steps involved in aldosterone biosynthesis, including expression of *CYP11B2 via* calcium/calmodulin-dependent protein kinases (16). Similarly, increased extracellular potassium concentrations induce cell membrane depolarization followed by activation of voltage-gated calcium channels and activation of calcium signaling (17).

**Figure 1:**
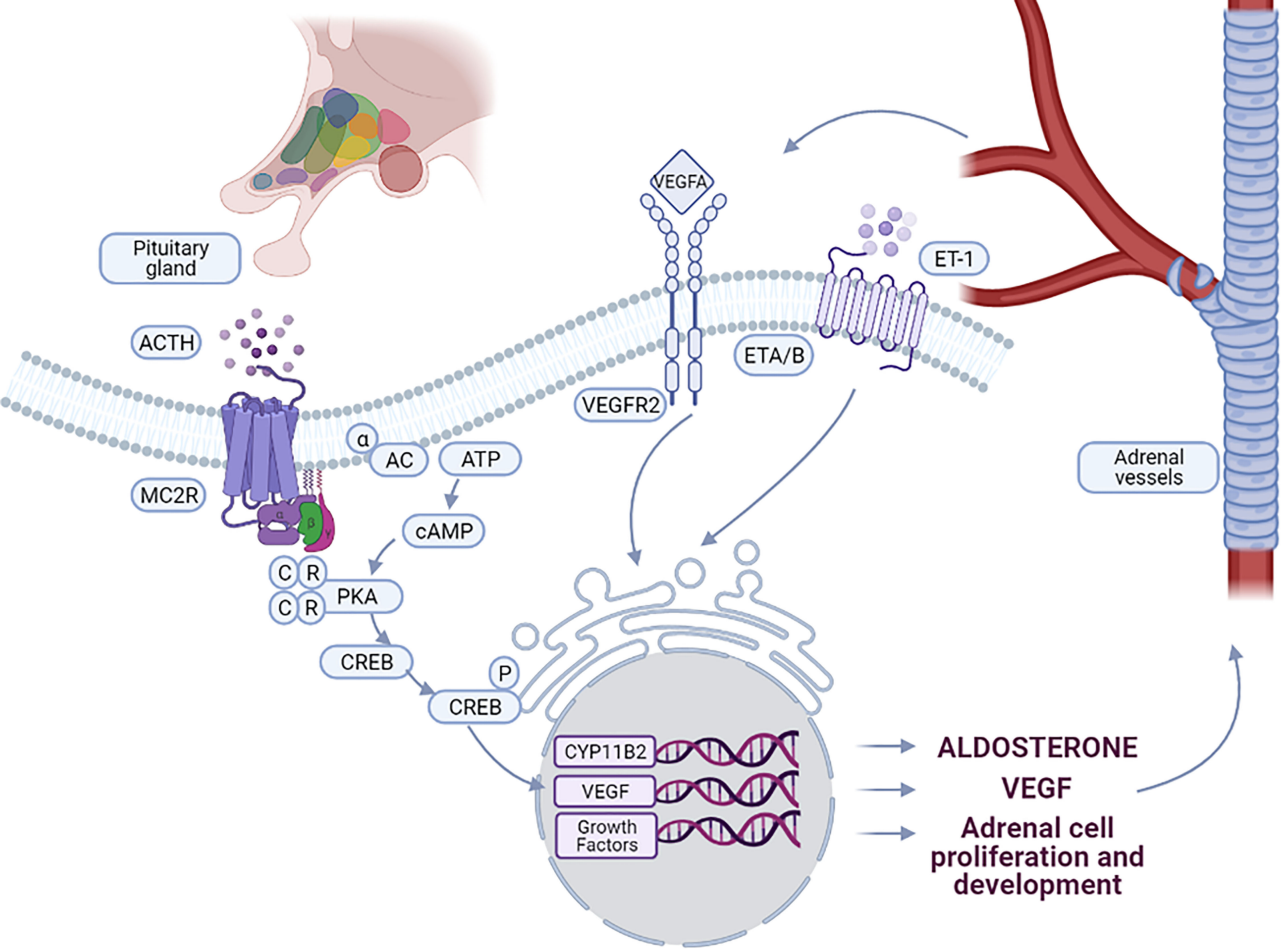
Interaction between vascular and steroidogenic cells in adrenal. ACTH, produced by the pituitary gland, controls the interactions between steroidogenic cells and vessels. Binding of ACTH to MC2R leads to the activation of PKA signaling pathway, stimulating CYP11B2 expression and aldosterone biosynthesis. ACTH stimulates also the expression of VEGF-A and specific growth factors by adrenal cells leading to vessel growth. By binding to VEGFR2, VEGF-A stimulates on one hand endothelial cell growth and on the other hand expression of growth factors involved in the maintenance and function of the adrenal cortex. Aldosterone biosynthesis is also induced by ET-1 through binding to the endothelin receptor ETA and ETB, expressed in the ZG of the adrenal cortex.

ACTH binds to its receptor (melanocortin type 2 receptor, MC2R) and activates adenylate cyclase (AC), with subsequent activation of downstream signaling pathways, in particular the cAMP-dependent protein kinase (PKA) pathway (**Figure 1**). PKA activates StAR (steroidogenic acute regulatory protein) either directly, or by increasing its expression *via* CREB (cAMP response element binding protein) phosphorylation, thus increasing the amount of cholesterol delivered to the inner mitochondrial membrane. The conversion of cholesterol to pregnenolone in the mitochondria is one of the principal limiting steps in steroid biosynthesis (18) catalyzed by the P450 side chain cleavage enzyme (P450scc or CYP11A1) which is located at the inner mitochondrial membrane. The P450scc catalyzes the 20α-hydroxylation, the 22-hydroxylation and the cleavage of the bond between C-20 and the C-22 of cholesterol to obtain pregnenolone (19). In addition, ACTH also increases the expression of other enzymes of the steroidogenic cascade, such as *CYP11A1*, increasing the amount of precursors for aldosterone biosynthesis (20, 21).

In addition to these endocrine regulatory loops, autocrine and paracrine regulation of aldosterone production has been described (**Table 1**). Different factors produced by steroidogenic cells, such as renin and AngII (22–24), epoxyeicosatrienoic acid (EET) or prostaglandin E2 (PGE2), may modulate aldosterone secretion though an autocrine mechanism (25–27). Components of the RAS have been detected in the adrenal, however, their role in adrenal function is still unclear (36). It has been proposed that the adrenal RAS could play a role in the control of aldosterone production under potassium stimulation (23). Interestingly, in wild-type mice, adrenal production of renin is observed during embryonic development while kidneys are immature (37), but down-regulated after birth. However, in specific mouse models, expression of renin is observed even in adult mice to compensate deficiency in proteins involved in the control of aldosterone biosynthesis. This is the case in Task3 potassium channel knock-out mice (38) or aldosterone synthase deficient mice (39), as well as in mast cell deficient mice under low salt diet (40). Deletion of Task3 in mice leads to low-renin salt-sensitive hypertension, with suppressed plasma renin and aldosterone biosynthesis that is not-suppressible by increasing salt intake (38). Furthermore, paracrine regulation of aldosterone production is mediated by factors released by components of the microenvironment both in normal human adrenals and adrenals with APA, i.e. chromaffin cells, endothelial cells, adipocytes, nerve fibers and immune cells (28, 33, 41, 42). Interestingly, serotonin (5-HT) released by perivascular mast cells is known to induce aldosterone production by activating the 5-hydroxytryptamine receptor 4 (5-HT4) expressed in ZG cells (34). Chromaffin cells and nerve fibers stimulate aldosterone production by secreting neurotransmitters (NT), including catecholamines and various neuropeptides (35). In particular, it has been shown recently that the neuropeptide substance P released by intraadrenal nerve fibres is able to regulate aldosterone biosynthesis in the human adrenal cortex by binding to neurokinin type 1 receptors (33). Cytokine C1q/TNF related protein and the adipokine leptin are also able *in vitro* to activate aldosterone production (30–32). ZG cells express leptin receptors, thus leptin released by adrenal adipocytes may have a direct effect on aldosterone production (43). Finally, endothelial cells secrete endothelin 1 (ET-1) which by binding to endothelin receptor type A and B (ET-A, ET-B) on adrenocortical cells can stimulate aldosterone production (29, 44, 45).

**Table 1:** Endocrine, paracrine and autocrine regulators of aldosterone biosynthesis.

Secretagogue	Localization	Effect	References
Local Renin Angiotensin System	Steroidogenic cells	Stimulation	(22–24)
Epoxyeicosatrienoic acid (EET)	Steroidogenic cells	Stimulation	(25)
Prostaglandin E2 (PGE2)	Steroidogenic cells	Stimulation	(26, 27)
Epinephrine, Norepinephrine	Chromaffin cells	Stimulation	(28)
Dopamine	Chromaffin cells	Inhibition	(28)
Endothelin 1 (ET-1)	Endothelial cells	Stimulation	(29)
Cytokine C1q/TNF related protein	Adipocytes	Stimulation	(30)
Leptin	Adipocytes	Stimulation	(31, 32)
Substance P	Nerve fibers	Stimulation	(33)
Serotonin (5-HT)	Immune cells	Stimulation	(34, 35)

## Vascularization of the adrenal cortex

As an endocrine organ, the adrenal gland is highly vascularized, allowing each endocrine cell to be in contact with an endothelial cell (3). Part of the arterial flow to both adrenal glands is provided by the ventral aorta. The remaining arterial supply is provided bilaterally by the renal artery, completed by the phrenic artery for the right adrenal and by a caudal branch of the aorta for the left adrenal (3, 4). In the middle of the medulla, the central vein is responsible for venous drainage. This vein merges with the vena cava in the right adrenal or the renal vein in the left adrenal. Three adrenal arteries are distinguished: the superior adrenal artery; the middle adrenal artery and the inferior adrenal artery.

From the capsule, two types of arteries emerge from the arteriolar capsular plexus and enter the cortex and medulla: 1) The arteriae medullae, responsible for medulla arterial supply after passing directly through the adrenal cortex; and 2) The arteriae cortices, that arise directly from the capsule plexus, form an anastomotic network in the ZG, then cross the ZF as longitudinal capillary sinusoids between the columns of ZF cells (3, 4). The vasculature of the adrenal gland is composed of fenestrated sinusoids (46) that are highly permeable to fluids and small molecules. This facilitates the supply in nutrients, oxygen and cholesterol to the gland and the secretion of steroid hormones into the blood flow.

The widely branched capillary bed of the adrenal cortex strongly suggests an interaction between endothelial cells (ECs) and adrenal epithelial cells (4, 47). Indeed, ACTH controls the coordinated development of vessels and endocrine cells (**Figure 1**). The interaction between adrenocortical cells and endothelial cells enables a coordinated development of the vascular network with the proliferation of adrenal cells and organ growth. Endocrine glands are characterized by the high expression of vascular endothelial growth factor (VEGF) even in adults, regardless of the absence of active angiogenesis. In this context, the role of VEGF-A, whose expression is controlled by ACTH, is to maintain a high density of stable fenestrated microvessels (48). The combined secretion of angiogenic factors by endocrine cells and trophic factors by endothelial cells makes it possible to maintain a “symbiosis” between these cellular compartments. Regulation of adrenal vascularization and growth must be coordinated to ensure that the cortical mass has appropriate vascular support essential for both growth of the adrenal cortex and its endocrine function (49).

## Regulation of vascularization in the adrenal cortex

Maintenance of the vascularization of the adrenal cortex and the regulation of blood flow by vasoconstriction involves different signaling pathways. AngII plays an important role in the regulation of blood pressure through its direct action on vasoconstriction (50). Interestingly, in the adrenal, vasodilatation or vasoconstriction may occur depending on the levels of AngII. Low concentration of AngII induces vasodilatation, *via* AT2R activation, production and release of nitric oxide (NO) by endothelial cells, whereas increased concentration of AngII leads to vasoconstriction due to activation of smooth muscle AT1R, resulting in decreased adrenal blood flow (51). ACTH, on the other hand, plays a role in the development and maintenance of this vascularization, and regulates blood flow to the adrenal gland through the release of vasorelaxant agents by adrenocortical cells such as metabolites of arachidonic acid (EETs) (52), but also through the release of histamine and serotonin by adrenal mast cells, factors modulating the tonicity of adrenal arterioles (53), indirectly influencing the production of steroids. Also, in response to ACTH, adrenal cortex cells secrete Thrombospondin-2 (TSP2), a large matricellular protein (54). It has been shown that TSP2 may act as an inhibitor of angiogenesis. *In vitro*, TSP2 inhibits the migration of capillary endothelial cells and, *in vivo*, neovascularization (55). It has been also shown that NO induces angiogenesis *via* the suppression of TSP2 expression, confirming the anti-angiogenic role of TSP2 (56). In addition, TSP2 may mediate ACTH-dependent centripetal adrenocortical cell migration (57). However, in mice lacking TSP2 no alterations in adrenal cortex morphology were observed (58, 59).

In the adrenal gland, ACTH also stimulates the release of VEGF and stabilization of its mRNA by the HuR protein (60). Conversely, the suppression of ACTH by dexamethasone in mice induces a progressive decrease in the expression of VEGF in the cells of the adrenal cortex and the regression of the vascularization (3). Interestingly, studies have also shown the role of mast cells in the development and maintenance of vascularization and in vasoconstriction. Mast cells are important cells in the immune system that originate from hematopoietic stem cells, which secrete serotonin, chondroitin, histamine and protease (61). Resident adrenal mast cells modulate the blood flow by the release of histamine and serotonin (5-hydroxytryptamine; 5-HT) (62). These cells are also a source of angiogenic factors including VEGF, fibroblast growth factor (FGF) 2, transforming growth factor β (TGF-β), tumor necrosis factor-α (TNF-α) and interleukin 8 (IL8) (63). They also induce the expression of VEGF by the release of cytokines and growth factors (TNF-α, TGF-β, platelet-derived growth factor (PDGF), FGF2 and IL-6) (62). Growth factors and cytokines released by mast cells have the ability to modulate endothelial cell function by increasing the expression of E-selectin but also by stimulating other cells that facilitate angiogenesis such as fibroblasts, epithelial cells and macrophages (64). The activation of mast cells also allows an increase in microvascular permeability, which has proangiogenic effects following the release of histamine, prostaglandin D2, Leukotriene B4, Leukotriene C4, VEGF and platelet-activating factor (65). Finally, the adrenal gland is a richly innervated organ, allowing innervation of chromaffin cells of the adrenal medulla. This innervation is, therefore, under the control of the sympathetic nervous system and allows the innervation of the internal part of the adrenal cortex. The adrenal cortex is also thought to be innervated by sympathetic fibers originating from extra-adrenal neurons, which, together with the blood vessels form the subcapsular plexus (66).

Adrenal cortex and medulla develop from two separate embryological tissues: the medulla is derived from the neural crest, while the cortex develops from the intermediate mesoderm. During development, the human fetal adrenal (HFA) cortex that develops from the adrenogonadal primordium, is composed of two zones: the inner zone, referred to as the fetal zone (FZ) with high expression of steroidogenic enzymes and a smaller outer zone, called definitive zone (DZ) where expression of steroidogenic enzymes is lower (67). The FZ of the adrenal cortex is the principal site of VEGF synthesis and one of the most vascularized organs in the human fetus (68). This pattern of VEGF localization is consistent with the fetal zone being the most vascular compartment in the cortex and the primary site of adrenal cortical growth. Thus, VEGF may act as a local regulator of fetal zone vascularization. The fetal zone vasculature comprises an extensive sinusoidal plexus. In contrast, the vasculature of the definitive zone is composed of distinct arterioles that arise from terminal branches of the capsular arterial network and enter the gland along connective tissue trabeculae. Therefore, as the cortex grows, the bulk of neovascularization would be expected to occur in the fetal zone (69). This vascular arrangement results in centripetal blood flow from the capsule through the definitive zone and into the sinusoidal network of the fetal zone to eventually drain into the central vein. Angiogenesis is essential for the rapid growth of the HFA. In addition, the HFA requires the development of an extensive vascular system for the delivery of steroid hormone precursors to the gland and the secretion of hormone products into the peripheral circulation. Various factors are involved in the regulation of angiogenesis. The evaluation of the expression and regulation of angiogenic factors specific to vascular endothelial cells, VEGF family members, angiopoietins (Angs) 1 and 2 in HFA medium showed that these factors are expressed in the HFA and that ACTH regulates them in isolated HFA cortical cells, suggesting that these factors may be key local regulators of HFA angiogenesis (70). Thus, they can mediate the tropic action of ACTH, exerting parallel control over the vascular system. In particular, ACTH induces an altered balance in which Ang2 predominates over Ang1. In addition, the Ang2 protein is mainly localized in the periphery of the HFA (i.e. the DZ and the outer region of the FZ). Its expression has been restricted to vascular remodeling sites, and Ang2 has been proposed to make endothelial cells sensitive to angiogenic stimuli, such as VEGF-A and FGF-2 (71). Furthermore, Steroidogenic factor-1 (SF-1) and Ang2 were found to be coexpressed in early stages of HFA development (72). It is demonstrated that despite the role of SF-1 in adrenal development and function, it plays a crucial role also in its angiogenesis by activating the Ang2 gene promoter in HFA (73). By using chromatin immunoprecipitation (ChIP) microarrays, it has been shown that vascular remodeling is a mechanism regulated by SF-1 in adrenal development and tumorigenesis (72).

## Coordinated development of steroidogenesis and angiogenesis in the adrenal cortex by ACTH

The adrenal cortex is a highly plastic organ, in which environmental stimuli are translated to hormonal responses that can involve extreme tissue remodeling. Example of this is the ZG expansion observed under a low salt diet, which stimulates the renin-angiotensin system to promote aldosterone biosynthesis (74). On the other hand, endogenous or exogenous glucocorticoid excess, such as treatment with dexamethasone, leads to a major regression of the ZF and suppression of glucocorticoid production (75). Both these changes are reversible and may involve major modification of adrenal vascularization.

ACTH is the main hormone regulating the function of the zona fasciculata and zona reticularis and stimulating glucocorticoid biosynthesis; it also stimulates, to a lesser extent, aldosterone production by the ZG. In addition, ACTH when binding to its receptor MC2R in the adrenals also induces the adrenal production of factors affecting adrenal growth and its blood flow. Indeed, ACTH controls angiogenesis and vascularization in the adrenal gland by stimulating the intra-adrenal production of VEGF and the vaso-relaxant EETs. On the other hand, VEGF can act on adrenal cells by binding on VEGF receptors present on adrenocortical cells and stimulates aldosterone production. Vascularization and adrenal cortex development must be coordinated to ensure that adrenocortical cells have access to blood vessels as the adrenal growths.

The vasculature of the HFA is established by the eighth week of gestation when the adrenal is supplied by arteries from the descending aorta, and the capillary sinusoids within the gland form a continuum with the systemic circulation. This stimulation is mediated by specific angiogenic factors like VEGF (76). Shifren et al. (69) showed that the HFA cortex is highly vascularized, consistent with its function as an endocrine organ, and that the HFA cortex expresses VEGF, which may regulate cortical vascular development (76). ACTH increases the steady state abundance of mRNA encoding VEGF. This may suggest that VEGF expression and secretion by human fetal adrenal cortical cells are up-regulated by ACTH and factors that increase intracellular cAMP production. In the same study, the authors also demonstrated that forskolin and ACTH are able to stimulate VEGF expression and secretion by HFA adrenocortical cells. This suggests that adenylate cyclase and cAMP pathways are the main regulators of ACTH-dependent VEGF production. Therefore, ACTH induces steroidogenic enzymes, cortisol and aldosterone production and expression of different growth factors *via* the same pathway, suggesting that ACTH may coordinate vascularization, adrenocortical development and steroidogenesis in the adrenal gland.

## Cross-talk between aldosterone and the vascular system

In addition to AngII, extracellular potassium and ACTH, endothelin and VEGF have been shown to stimulate aldosterone production in a paracrine manner (28, 77). In particular, VEGF has been shown to stimulate aldosterone production indirectly by maintaining endothelial integrity but also directly by stimulating aldosterone synthase expression in ZG cells (77). The action of VEGF is either synergistic or independent of Ang II. Interestingly, in contrast to Ang II, VEGF does not increase the expression of StAR. The stimulatory role of VEGF is restricted to enhance aldosterone production, but does not modify cortisol biosynthesis in H295R adrenocortical cells (77). In addition, the inhibition of VEGF by overexpression of soluble fms-like tyrosine kinase-1 (sFlt-1) in rats is associated with reduced adrenal cortex vascularization (reduction of CD31 endothelial cell marker) that is accompanied by a reduction of aldosterone production (77). These results suggest that VEGF may have a role in aldosterone production independently of the RAS and may play a role in the autonomous overproduction of aldosterone in PA.

It has also been demonstrated that endothelial cell-conditioned medium stimulates aldosterone production in human adrenocortical H295R cells (78, 79). The interaction between endothelial and steroidogenic cells was demonstrated at the molecular level. *In vitro* studies in cultured human adrenocortical cells revealed that cytokines like IL-6 and ET-1, as well as NO produced by endothelial cells, are the main actors in this interaction (33, 42, 72). Adrenocortical cells express big ET-1, the precursor of endothelin, and its specific proteolytic enzyme endothelin converting enzyme, which generates the 21 amino acid peptide ET-1 (45, 80). Interestingly, ET-1 stimulates aldosterone biosynthesis in human and in rats (81). This action is mediated by the two endothelin receptor subtypes ETA and ETB expressed in the ZG of the adrenal cortex (33, 42).

On the other hand, aldosterone can act not only on renal epithelial cells to regulate blood pressure but also on vascular endothelial and smooth muscle cells. Indeed, activation of its receptor, the mineralocorticoid receptor (MR), has been shown to induce endothelial dysfunction, inflammation, remodeling, stiffening and atherosclerosis (82–88). In addition, it has been demonstrated in mice that aldosterone increases vessel density in response to ischemia, a phenomenon mediated by MR activation, AngII signaling and VEGF (89). Aldosterone also increases placental growth factor (PGF, a member of the VEGF family) expression in human atherosclerotic vessels, leading to inflammation and proliferation of vascular cells (90). Aldosterone may also up-regulates VEGF-A production in human neutrophils by activating PI3 kinases, ERK1/2, and to a lesser extent p38 MAPK pathways, suggesting that aldosterone has an active role on neovascularization (91).

## Interplay between vascular and hormonal components in primary aldosteronism

Adrenals with APA show increased nodulation and reduced vascularization in the peritumoral adrenal cortex, as well as ZG hyperplasia (92). In addition, different studies indicate that the structure and function of the adrenal cortex changes with age and it has been suggested that this may be a consequence of vascular dysfunction (93). Ageing is associated with an increase in adrenal nodulation in the general population, which may represent compensatory growth in response to ischemic changes due to localized atherosclerosis or hypertension (15). In addition, the adrenal cortex contains special structures, called aldosterone producing cell clusters (APCCs), in which somatic mutations have been identified in genes responsible for APA in normal subjects (94) and in adrenals with APA (12). It has been postulated that APCCs represent structures of autonomous aldosterone production. Interestingly, the number of APCCs increases with age (95), in parallel with a dysregulation of aldosterone production (93).

Mutations in different genes increase aldosterone production in PA, but additional mechanisms may contribute to increased cell proliferation and APA development. We have recently shown that retinoic acid receptor α (RARα) contributes to the maintenance of normal adrenal cortex structure and cell proliferation in mice, by modulating non-canonical Wnt signaling, extracellular matrix composition and angiogenesis. Dysregulation of this interaction may contribute to abnormal cell proliferation, creating a propitious environment for the emergence of specific driver mutations in PA (96). Indeed, RARα was identified as a central molecular network involved in adrenal nodulation in a transcriptome study comparing 48 APA and 11 control adrenals. Inactivation of Rarα in mice induced a major structural disorganization of the adrenal cortex in both sexes, with increased adrenal cortex size in female mice and increased cell proliferation in males. These changes were associated with abnormalities of vessel architecture and extracellular matrix. At the molecular level, Rarα inactivation led to decreased expression of components of the non-canonical Wnt signalling pathway, with decreased expression of *Wnt4*, *Tcf3*, *Lef1*, without affecting the canonical Wnt pathway nor PKA signaling. Rarα inactivation also reduced the expression of VEGF-A, while other angiogenesis factors such as VEGF-C and Hif1α were not affected. In contrast, the expression of components of the extracellular matrix like fibronectin 1, microfibrillar associated protein 2 and 5 (Mfap2 and Mfap5) and collagen 3α1 were increased. Altogether, these data highlight the important role of the interplay between the vascular and hormonal components in the adrenal cortex and suggest that alterations affecting this interplay, such as modifications of the extracellular matrix composition, may contribute to the disorganization of the adrenal cortex by modulating adrenocortical and vascular cell migration (96).

As observed in normal adrenals, it is expected that endothelin secreted by adrenal vessels and its signaling pathway in steroidogenic cells can stimulate aldosterone production and may have a role in its autonomous overproduction and by consequence contribute to the development of PA (80). Rossi et al. showed that selective endothelin receptors ETA and ETB antagonists lowered blood pressure in patients with PA and high to normal renin hypertension. Interestingly, in PA patients, these selective antagonists induced also a decrease in aldosterone biosynthesis (97, 98). However, in peripheral blood samples, ET-1 levels were similar in control subjects and in patients with APA; similar expression of prepro-ET-1, the endothelin-converting enzyme and the endothelin receptors ETA and ETB was found in APA and in normal adrenal gland (99). Moreover, ET-1 was demonstrated to have the same stimulatory effect as AngII on aldosterone production in APA (97). However, ET-1 receptors were found to be partially downregulated in APA in another study (45). These observations suggest that the endothelin signaling pathway in adrenocortical cells may play a role on aldosterone biosynthesis in APA and normal adrenals, but that this system is not crucial in the pathogenesis of this disease.

Recently, we have investigated the relationship between different signaling pathways and components of the microenvironment in adrenals with APA. We have applied multiplex immunofluorescence and multispectral image analysis to investigate the colocalization of proteins involved in aldosterone (CYP11B2) and cortisol (CYP11B1/CYP17A1) biosynthesis, markers of Wnt/β-catenin (β-catenin) and ACTH/cAMP/PKA (MC2R, pCREB) signaling, as well as paracrine pathways of the tumor microenvironment (Tryptase, S100) and vascularization (CD34) (100). Our results show a dense vascularization in APA, which is independent of the somatic mutation status of the tumor. Although the vascular surface was similar in areas expressing aldosterone synthase and areas not expressing aldosterone synthase in APA, VEGF-A expression analyzed by RT-qPCR in three APA was higher in areas expressing aldosterone synthase. This difference may be explained by the presence of perivascular mast cells, which was higher in areas positive for aldosterone synthase expression, which are involved in the maintenance of angiogenesis; alternatively, activation of the ACTH/cAMP pathway *via* MC2R may be involved, as MC2R was highly expressed in these same regions.

Vascularization and angiogenesis were also studied in other types of benign adrenocortical adenomas as well as in malignant adrenocortical tumors (ACC). Whether it was a benign or a malignant tumor, carriers of adrenocortical tumors showed higher circulating VEGF levels in comparison with healthy subjects (101, 102). Interestingly, VEGF expression was shown to be very high in patients with adrenocortical carcinomas and higher in APA in comparison to non-functional adenomas (103). Despite the high expression of VEGF, APA did not present higher vascular density than normal adrenals, but remarkably APA presented higher vascular density than non-functional adenomas, cortisol producing adenomas and adrenal cortical carcinomas (103). The same study also demonstrated that in APA, vascular density is positively correlated to aldosterone levels and negatively correlated to plasma renin activity. These results suggest that angiogenesis and the functional status of adrenocortical tumors are closely associated.

## Conclusions

In conclusion, the coordinated interaction between steroidogenic and endothelial cells plays a crucial role in adrenal development and function and allows adrenal cortex remodeling in response to different physiological stimuli, such as modifications of sodium diet or stress response. Key players in this interaction appear to be ACTH and VEGF, which cross-talk to regulate hormone biosynthesis and vessel growth (**Figure 1**). Alterations in this interaction or factors affecting adrenal cortex vascularization may modify adrenocortical cell growth and promote cell proliferation and nodule formation, creating a propitious environment for the occurrence of somatic mutations in genes involved in the development of PA. Further studies will allow deciphering how this interplay may be altered in physiological or pathological conditions.

## Author contributions

All authors listed have made a substantial, direct, and intellectual contribution to the work and approved it for publication.

## Funding

The laboratory of Dr. Maria-Christina Zennaro is supported through institutional funding from INSERM, by the Agence Nationale de la Recherche (ANR-18-CE93-0003-01) and the Fondation pour la Recherche Médicale (EQU201903007864).

## Conflict of interest

The authors declare that the research was conducted in the absence of any commercial or financial relationships that could be construed as a potential conflict of interest.

## Publisher’s note

All claims expressed in this article are solely those of the authors and do not necessarily represent those of their affiliated organizations, or those of the publisher, the editors and the reviewers. Any product that may be evaluated in this article, or claim that may be made by its manufacturer, is not guaranteed or endorsed by the publisher.
